# Patterns and correlates of two-year changes in depressive symptoms for autistic adults

**DOI:** 10.3389/fpsyt.2024.1461704

**Published:** 2024-12-03

**Authors:** Shuting Zheng, Cristan Farmer, Julie Lounds Taylor, Ryan Adams, Lindsay Olson, Somer Bishop

**Affiliations:** ^1^ Department of Psychiatry and Behavioral Sciences, University of California, San Francisco, San Francisco, CA, United States; ^2^ Neurodevelopmental and Behavioral Phenotyping Service, National Institute of Mental Health, Bethesda, MD, United States; ^3^ Department of Pediatrics, Vanderbilt University Medical Center, Nashville, TN, United States; ^4^ Department of Pediatrics, Cincinnati Children’s Hospital Medical Center, Cincinnati, OH, United States

**Keywords:** depression, longitudinal trajectories, employment, school, income level

## Abstract

**Background:**

Autistic adults are at elevated risk for depression. However, longitudinal data on the trajectory of depressive symptoms and its associated factors in autistic adults are scarce.

**Methods:**

A community sample of 315 autistic adults participated in a two-year longitudinal study from the beginning of (March 2020) to the recovery from the COVID-19 pandemic (March 2022). They provided five waves of data on self-reported depressive symptoms and sociodemographic and life circumstances information.

**Results:**

Multilevel model results showed that autistic adults reported large between-individual variability in self-reported depressive symptoms, and on average, they experienced an increase (i.e., worsening) in self-reported depressive symptoms over the two years of the study. Autistic adults with a depression history and lower annual household income reported higher levels of depressive symptoms. More importantly, autistic adults reported lower depressive symptoms when they were engaged in work or school, and those who had higher levels of depressive symptoms at the start of the study were more reactive to changes in work or school participation.

**Conclusions:**

Findings from the current study have implications for potential venues of depression treatment in autistic adults around promoting employment/education, providing symptom monitoring, and addressing mental health disparities for those with lower incomes.

## Introduction

While research during the past decade clearly shows elevated rates of depression among autistic adults (1, 2), little is known about the course of depressive, or factors associated with symptom changes, in this population. Existing research on risk and protective factors associated with depression in autism has relied on cross-sectional or short-term longitudinal (e.g., a few months apart) data (3–5). However, longer-term studies with more data points are needed to delineate depressive symptom trajectories and identify individual-level factors associated with symptom changes over time. Such data have the potential to elucidate etiological mechanisms underlying depression in autism, and inform modification and delivery of more targeted interventions. Thus, the goal of the current study was to understand individual depression symptom trajectories in autistic adults, including the extent to which these trajectories are influenced by sociodemographic factors and changes in life circumstances that have been previously associated with depressive symptoms in the general population.

It is well established that social determinants of health, such as socioeconomic status (SES), influence depression and access to treatment in the general population (6, 7). Similarly, SES variables have been found to impact service receipt among autistic people (8), including depression treatment access (9), but their impact on depression has not been directly investigated in this group. Sex assigned at birth and diverse gender identity are other sociodemographic factors related to differential rates of depression in the general population, with females assigned at birth and gender-diverse individuals at increased risk compared to males assigned at birth (10). Emerging evidence also show that autistic females are diagnosed with depression at a higher rate than autistic males (11, 12), but, little is known about how sex assigned at birth or diverse gender identity might account for variability in longitudinal trajectories of depression in autism. Previous mental health history is another significant risk factor for later depression, with high reoccurrence in the face of life stressors (13). Recent studies on the early impact of the COVID-19 pandemic and increased vulnerability among autistic individuals with higher levels of internalizing symptoms suggests that previous mental health history may be also be an important risk factor for autistic adults (14–17). However, the short window of data collection may limit generalizability of these findings beyond the initial response to the pandemic.

Years of research with non-autistic adults have shown that job loss and prolonged unemployment are associated with worsening mental health (18), while being in romantic relationships could have a buffering effect (19). This has significant implications for depression in autism, as many autistic adults face significant challenges in these important aspects of life (20), including low rates of post-secondary education, high rates of unemployment and underemployment (21), and low rates of social participation and relationships during young adulthood (22). In fact, in our initial follow-up of the current sample, which occurred about ten weeks into the COVID-19 pandemic, we found that 38% of adults had experienced job loss. Furthermore, those who experienced job loss experienced significant deterioration in depression compared to those with stable employment (23), consistent with findings from another study on employment changes in autistic individuals during the pandemic (24).

The current study collected five waves of data from March 2020 to March 2022, covering the two years from immediately before the beginning of the COVID-19 pandemic, through the initial recovery from the pandemic. We collected data on sociodemographic variables and changing life circumstances during this time and investigated how they might be related to changes in depressive symptoms. The COVID-19 pandemic ushered in a time of relatively frequent employment changes (25) and relationship distress (26), allowing us to capture a degree of life circumstance change that would typically take much longer to observe. We set out to address two main research questions:

1. Among autistic young adults, what was the course of depression symptom changes from pre-pandemic over the two years that followed?
2. Are sociodemographic variables and changes in life circumstances during the study period associated with trajectories of depressive symptoms?

## Methods

### Participants and procedures

A community sample of 700 eligible SPARK research match (27) registrants was contacted at the beginning of March 2020 to participate in a study about depression and depression service receipt among autistic adults (9). All participants met the following inclusion criteria: (1) aged between 18 and 35 years at the initial survey wave; (2) diagnosed with autism spectrum disorder (ASD) before 18 years of age (mean age of autism diagnosis: 8.74 years old [SD=4.63]); and (3) could legally provide consent for themselves and independently complete self-report questionnaires. Within the first two weeks of the study launch, 315 legally independent adults completed the full survey, exceeding the recruitment goal of 300. Given that we had no way to directly confirm diagnosis, we limited our sample to individuals who reported receiving a diagnosis of ASD during childhood in order to account for the large variability in diagnostic practices for children versus adults. Further, given the extreme phenotypic heterogeneity among adults with autism, partially as a function of the age of diagnosis (28, 29), we believed that limiting to a childhood-diagnosed sample would improve the interpretability and clinical utility of the findings.

**Figure 1:**
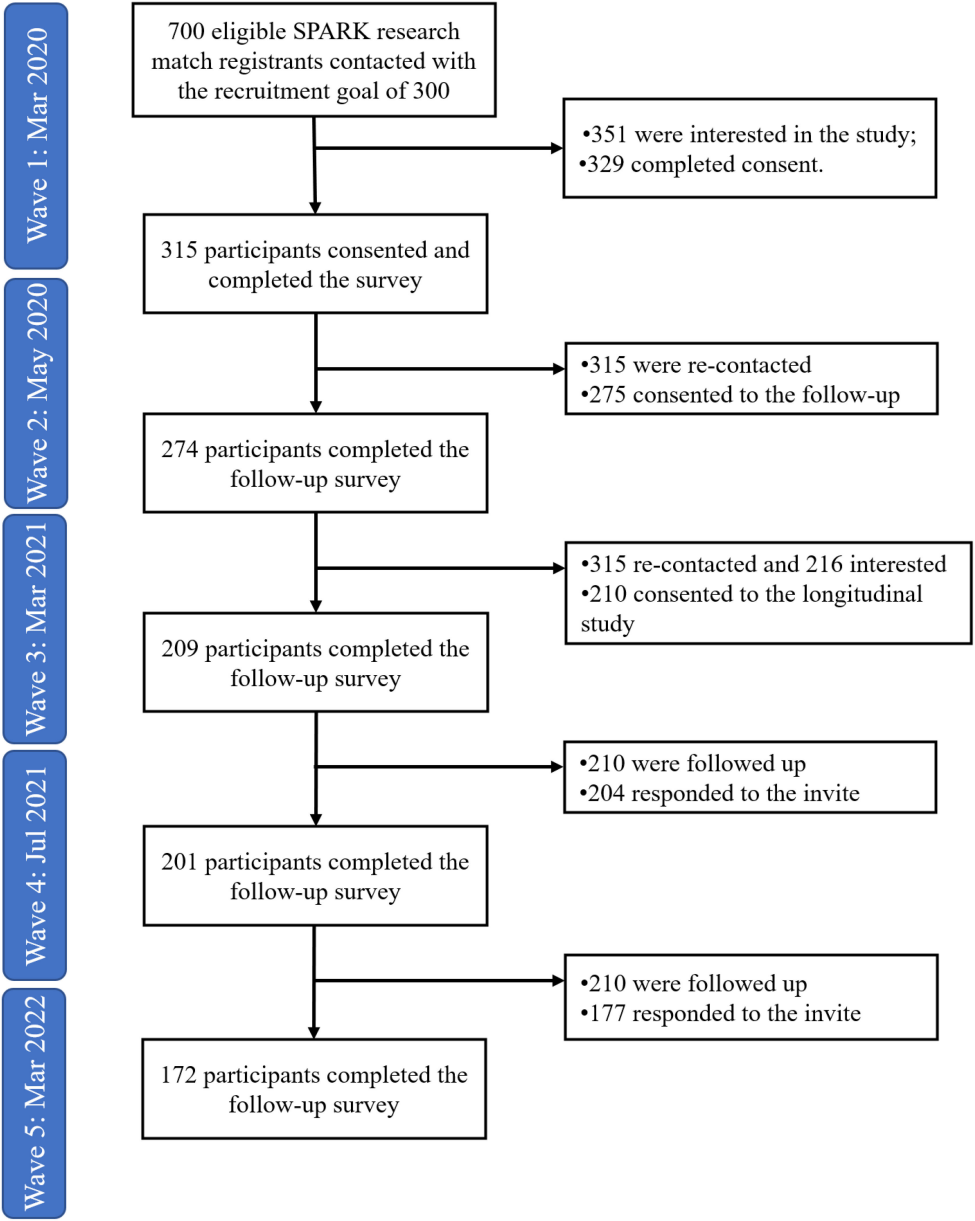
Participant flowchart across timepoints.

While not initially conceived as a longitudinal study, we took the opportunity to conduct a follow-up survey in mid-May 2020 (274 out of 315 participants completed Time 2 survey). These first two waves of data collection were administered through the SPARK online survey workflow. Then, given our interest in continued follow-up of the cohort, we initiated the SPARK research match hand-off process, which allowed us to begin managing data collection locally within the research team. Of the 315 participants who completed the Time 1 survey, 210 provided consent to be followed longitudinally by our team. Surveys were sent in March 2021 (209 out of 210 completed the Time 3 survey, July 2021 (203 out of 210 completed the Time 4 survey), and then again in March 2022 (172 out of 210 completed the Time 5 survey), totaling five timepoints over two years. The timing of data collection waves was chosen based on the availability of funding. For each wave of data collection, participants completed surveys within a 2-week period. See **Figure 1** for the flowchart of participant recruitment and retention. Participants who dropped out did not differ from those who remained in the study in any main characteristics of interest at baseline (i.e., age, depression history, baseline depressive symptom, sex assigned at birth and diverse gender identity, race, rates of employment and school enrollment, relationship status or education level). Participants received a US$25 e-gift card for the completion of surveys at each timepoint. All study procedures were approved by the Internal Review Board at the authors’ institutions.

The study was reviewed and approved by the Participant Access Committee consisting of community and scientific advisors at the SPARK research match registry (https://sparkforautism.org/portal/page/participant-access-committee/). Participant feedback and suggestions for the study (free-text question: “You can share any feedback or suggestions you might have with us below.”) were collected at each timepoint to inform necessary modifications of subsequent data collection waves (e.g., regarding measure selection, as described below).

### Measures

#### Socio-demographics

Participants’ demographic information was collected through the study surveys across timepoints. At baseline, participants reported sex assigned at birth (male vs. female with male as the reference category for analysis), and history of depression (with vs. without depression history, with no depression history as the reference category). Household income level was collected at the last timepoint using a multiple-choice question: “What is your annual household income?”. Response options were: Less than $20,000, $21,000 - $35,000, $36,000 - $50,000, $51,000 - $65,000, $66,000 - $80,000, $81,000 - $100,000, $101,000 - $130,000, $131,000 - $160,000, Over $161,000. To increase the interpretability of model estimates, the midpoint value of each category in thousands was used in the current analyses (e.g., $28K for category $21,000 - $35,000), while $20K was used for the Less than $20,000 and $161K for the Over $161,000. The income variable was used to approximate the participant’s financial resources. Gender identity was collected at four out of five timepoints (not collected at Time 2). Given the limited number of individuals reporting changes in gender identity during the study period (n=18), we derived a time-invariant variable for diverse gender identity to represent participants who had ever reported diverse gender identity during the study period (sex assigned at birth =gender identity [reference category] vs. sex assigned at birth ≠gender identity).

#### Life circumstances

Life circumstances, including employment status, school enrollment, and romantic relationship status (single [reference category] vs. in a romantic relationship [including dating and married]) were collected at each timepoint. Because our sample was between the ages of 18 and 35 years, some participants were still in school at baseline (eight were in high school, 84 were in post-secondary education programs). It is common to consider participation in both educational and vocational activities as indices of daytime activities, as young adults who are not working may be full-time college students (8). Therefore, in the current analysis, school enrollment and employment status were synthesized to a binary variable “work/school activity”: neither working nor in school (reference category) vs. any educational/work activity. To ensure that individuals who were still in high school did not impact the findings, given that their social contexts might be quite distinct from the rest of the sample, we conducted sensitivity analyses with the eight high schoolers at baseline excluded. The patterns of results did not change, and these eight participants were retained in the analyses presented in this manuscript.

#### Depressive symptoms

The Depression Anxiety Stress Scale-21 (DASS-21) is a short version of the DASS-42. Based on participants’ feedback that the DASS-42 was too long, we switched to the DASS-21 for the later three waves of data collection. It contains 21 items (on a scale of 0–3) describing symptoms related to depression, anxiety, and stress, with seven items for each subscale. In a psychometrics study of the DASS-21 in a sample of autistic individuals aged 16 to 46 years old with IQ over 70, the DASS-21 showed good reliability and validity (30). For the current analyses, we focused on the depression subscale. Based on the DASS manual, the raw sum of the subscale were multiplied by 2; the recommended cut-off for clinical concern is a score of 14 and above (moderate to extremely severe concern) (31). The depression score for each timepoint was created using the seven items included on the DASS-21 (regardless of whether the DASS-42 or DASS-21 was administered). In the current sample, the DASS-21 Depression Subscale showed good internal consistency, with Cronbach’s alpha ranging from 0.92 to 0.94 across the five timepoints.

### Statistical analyses

Sample characteristics were described across timepoint. Bivariate associations of the independent variables were calculated. Frequencies of changes in life circumstance variables (i.e., work/school activity and romantic relationship status) between consecutive timepoints were reported. Descriptive statistics for depressive symptoms at each timepoint were provided.

#### Multilevel models

With the longitudinal repeated measure design, self-report depressive symptom scores across timepoints were nested within individual participants. We employed multilevel regression to capture within- (Level 1) and between-individual (Level 2) variance. Time was coded as elapsed months from baseline, given the unequal spacing of study intervals (2 months, 12 months, 16 months, and 24 months from baseline). Age at baseline was included as a control variable for all models. The model with repeated timepoint nested within individuals and no predictors was estimated to calculate intra-class correlation (ICC) to describe the proportion of variance explained by between-individual differences. Parameter estimates of fixed effect were reported with 95% confidence interval and t-statistics (threshold of uncorrected *p* value is set at.05) to represent the effects of predictors on depressive symptom changes. Random effects estimates (i.e., standard deviations) and their correlations were reported to understand between individual variability of the depression symptom trajectories and their associations with time-varying variables. Models are estimated with Restricted Maximum Likelihood estimation.

To address Research Question (RQ) 1, a multilevel model with both fixed effect and random effect of linear trend of time was first fitted to estimate the overall trend of depressive symptom changes. Then, the fixed effect and random effect of quadratic term of time were added to the model with linear effect. The two models were estimated using Full Information Maximum Likelihood and compared by likelihood ratio test to examine if adding the quadratic term of time improved the model fit: if not, the linear model was retained as the initial model for later model building; otherwise, later model building was based on the model with quadratic effect of time.

To address RQ 2, time-invariant sociodemographic variables (i.e., sex assigned at birth and diverse gender identity, depression history, household income level) and their interaction with time were included as fixed effects to examine their associations with between-individual differences in the overall levels and changes in depressive symptoms. Life circumstance variables (i.e., work/school activity and romantic relationship status) were included as time-varying variables with both fixed and random effects to examine their associations with changes in depressive symptoms over time. Moreover, because the study spanned different phases of the pandemic, the interaction effects of life circumstances variables and time were included as a fixed effect in their respective models to examine whether the effects of life circumstances on depressive symptom changes differed as a function of time. Each variable was included in separate models to test their independent association with longitudinal changes in depressive symptoms. The assumptions of the normality of Level-1 residuals and Level-2 random effect were checked via visual inspection of density plots and Q-Q plots for the models (see **Supplementary Material Figures S1, S2**).

The multilevel models were fitted with *lme4* and *nlme* packages in R 4.1.0.

## Results

At baseline (March 2020), participants had a mean age of 26.33 years (SD=4.64, Range 18.1 to 35.9 years), with 48% females assigned at birth, 80% non-Hispanic White, 58% reporting an annual household income of $36,000 or lower, and 65% reporting a history of depression diagnosis (see **Table 1** for detailed sample descriptives across timepoints). For life circumstances at baseline, 52% reported paid employment and 29% were enrolled in school programs (67% involved in work and/or school activity), while 37% were in a romantic relationship. **Supplementary Table S1** shows the number of cases that changed work/school activity (ranging from 12% to 16%) and romantic relationship status (ranging from 5% to 10%) between timepoints. Bivariate correlations between the independent variables were nominal (smaller than 0.2), with the exception that income level and work/school activity status were correlated at 0.37.

**Table 1:** Descriptive statistics across timepoints.

		Baseline (N=315)	Time 2 (N=274)	Time 3 (N=209)	Time 4 (N=201)	Time 5 (N=172)
Lifetime Depression Diagnosis Baseline	Never Diagnosed	109 (35%)				
	Ever Diagnosed	206 (65%)				
Sex Assigned at Birth	Male	165 (52%)	139 (51%)	105 (50%)	101 (50%)	87 (51%)
	Female	150 (48%)	135 (49%)	104 (50%)	100 (50%)	85 (49%)
Gender Identity	Male	163 (52%)		102 (49%)	96 (48%)	87 (51%)
	Female	133 (42%)		87 (42%)	85 (42%)	73 (42%)
	Non-binary	18 (6%)		20 (9%)	20 (10%)	12 (7%)
Race	White	259 (82%)	226 (83%)	173 (83%)	167 (83%)	142 (83%)
	African American	11 (4%)	9 (3%)	7 (3%)	7 (4%)	6 (3%)
	Asian	4 (1%)	3 (1%)	2 (1%)	2 (1%)	1 (1%)
	Multi-racial	36 (11%)	32 (12%)	23 (11%)	21 (10%)	20 (12%)
	Other	5 (2%)	4 (1%)	4 (2%)	4 (2%)	3 (2%)
Ethnicity	Non-Hispanic	287 (91%)	248 (90%)	189 (90%)	182 (91%)	158 (92%)
	Hispanic	28 (9%)	26 (10%)	20 (10%)	19 (9%)	14 (8%)
Household income Level	Less than $20,000					62 (36%)
	$21,000 - $35,000					37 (22%)
	$36,000 - $50,000					23 (13%)
	$51,000 - $65,000					11 (6%)
	$66,000 - $80,000					15 (9%)
	$81,000 - $100,000					9 (5%)
	$101,000 - $130,000					7 (5%)
	$131,000 - $160,000					1 (1%)
	Over $161,000					6 (4%)
Highest Education Level	High school and below	121 (38%)		69 (33%)	66 (33%)	60 (35%)
	Above high school	194 (62%)		140 (67%)	135 (67%)	112 (65%)
Relationship Status	Single	199 (63%)	175 (64%)	137 (66%)	128 (64%)	120 (70%)
	In a Relationship	116 (37%)	99 (36%)	72 (34%)	73 (36%)	52 (30%)
Employment Status	No paid employment	152 (48%)	140 (51%)	103 (49%)	96 (48%)	87 (51%)
	Paid employment	162 (52%)	134 (49%)	106 (51%)	105 (52%)	85 (49%)
School Enrollment	Not in school	222 (71%)	198 (72%)	152 (73%)	155 (77%)	132 (77%)
	Enrolled in school	92 (29%)	76 (28%)	57 (27%)	46 (23%)	40 (23%)
Work/School		212 (67%)	174 (64%)	141 (67%)	130 (65%)	104 (60%)
DASS-21 Depression Score		Mean (Standard Deviation)				
		12.90 (11.81)	12.18 (11.48)	13.95 (11.74)	14.80 (12.48)	15.51 (12.26)
		Medium (Interquartile Range)				
		10 (18)	8 (16)	10 (18)	14 (22)	14 (20)
Age in Years		Mean (Standard Deviation)				
		26.3 (4.6)	26.6 (4.6)	27.1 (4.5)	27.5 (4.6)	28.4

**Figure 2:**
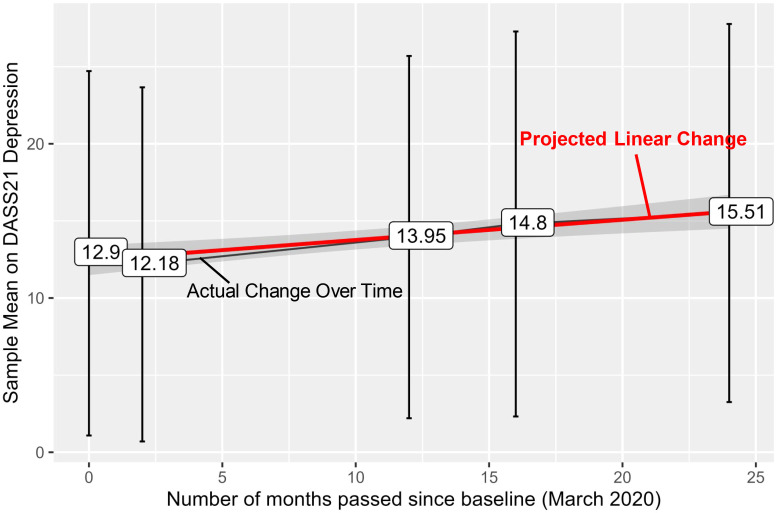
Sample means across timepoint with a linear trend line. The trend line shows that the sample reported an increase over the two years (2.61-point raw increase on average, 20% increase compared to baseline). The black line represents the sample average of self-reported depressive symptoms at each timepoint, with the labels representing the actual means and the whiskers representing ± 1SD. The red line represents projected linear change based on the sample average across the time.

**Figure 2** presents the average levels (whiskers representing standard deviation) of depression symptoms for the sample over time, and a projected linear change line indicating an initial small decrease in depressive symptoms in May 2020, followed by an increase over the course of the study (see **Supplementary Figure S3** for density plots of depressive symptoms of the sample across time points, with left-skewed distributions). Autistic adults reported median depressive symptom scores on the DASS-21 of 10 (IQR=18) at baseline, 8 (IQR=16) at Time 2, 10 (IQR=18) at Time 3, 14 (IQR=22) at Time 4, and 14 (IQR=20) at Time 5. The proportion of autistic young adults reporting clinical range depressive symptoms (moderate and above, score>=14) increased significantly over time from 43% in March 2020 to 55% in March 2023 (χ²_(df=16)_=32.39, *p*=0.009; see **Supplementary Figure S4** for stacked bar graph of DASS-21 depression categories across timepoint).

The ICC of the multilevel model with no predictors indicated that 73% of the observed variance in DASS-21 depression scores was attributable to between-individual differences and 27% was attributable to within-individual changes.

### Longitudinal trajectories of depressive symptoms

Both the linear and quadratic terms for time were estimated in the multilevel models to depict the longitudinal trajectories of depression. The likelihood ratio test showed that estimating a quadratic trajectory did not meaningfully improve model fit (ΔAIC=6.2, *p*=0.777; see **Table 2** for parameter estimates for both models). Therefore, the model with the linear main effect of time was kept for later model building. Model results showed that, on average, autistic adults in this sample reported depressive symptoms scores of 12.57 (a cut-off of 14 corresponds to moderate levels of depressive symptoms; 31) at baseline, with increases of 0.15 points (95% confidence interval [CI]: 0.09-0.20) in depressive symptom scores per month. This corresponds to a 3.6-point increase [28.6% increase compared to model-indicated baseline level] over two years, placing the sample average above the clinical cut-off on the DASS Depression scale by March 2022 (Timepoint 5). The random effects indicated large between-individual variability in baseline levels of depressive symptoms and some variability in the rates of change over time, warranting further investigation of whether individual characteristics account for the observed variability. The correlation between random intercept and random slope was nominal (-0.09).

**Table 2:** Longitudinal trajectory of depressive symptom model results.

	*Linear Growth Model*		*Quadratic Growth Model*		
Fixed Effects	Estimate (95% CI)	*t value (p)*	Estimate (95% CI)	*t value (p)*	
Intercept	10.64 [3.69, 17.58]	3.01 (.003)	10.76 [3.82, 17.70]	3.04 (.003)	***Likelihood Ratio Test:*** ΔAIC=6.2, *p*=0.777
Age at baseline	0.07 [-0.19, 0.33]	0.56 (0.577)	0.072 [-0.19, 0.33]	0.54 (0.588)	
Time	***0.15 [0.09, 0.20]***	5.25 (<.001)	0.10 [-0.04, 0.24]	1.41 (0.159)	
Time^2^			0.002 [-0.003, 0.008]	0.67 (0.503)	
Random Effects	Standard Deviation		Standard Deviation		
Intercept	10.31		10.21		
Time	0.27		0.33		
Time^2^			0.003		
Residual	5.53		5.52		

Bold and italicize values are statistically significant: p value <.05.

### Time-invariant sociodemographic variables

Depression history and income level were associated with depressive symptom level at baseline (i.e., the intercept) but not with the rate of change (their interaction effect with time was close to 0). Model estimates suggested that adults with a history of depression diagnosis scored on average 7.54 points (95% CI: 5.08-10.00) higher on the DASS-21 than those no previous depression diagnosis. Additionally, for every $10k increase in annual household income, autistic adults scored one half point lower on depressive symptoms. Sensitivity analyses using the original income categories as ordinal variables in the model also found a significant effect of income level. Neither the main effect of sex assigned at birth or diverse gender identity, nor their interactions with time, were statistically significantly associated with depressive symptom trajectories. The correlations between random intercepts and random slopes of the respective models were small ([-0.09, -0.14]). **Table 3** shows model estimates with each time-invariant variable and their interaction with time.

**Table 3:** Results from multilevel models with time-invariant variables.

	Sex Assigned at Birth		Gender Diversity Status		Depression History at Baseline		Income Level (in $1,000)	
Fixed Effects	Estimate (95% CI)	*t value (p)*	Estimate (95% CI)	*t value (p)*	Estimate (95% CI)	*t value (p)*	Estimate (95% CI)	*t value (p)*
Intercept	9.89 [2.74, 17.04]	2.70 (0.007)	9.76 [2.80, 16.71]	2.75 (0.006)	6.90 [0.22, 13.57]	2.02 (0.044)	12.08 [2.33, 21.83]	2.43 (0.016)
Age at baseline	0.08 [-0.18, 0.35]	0.64 (0.523)	0.09 [-0.17, 0.35]	0.70 (0.483)	0.03 [-0.22, 0.27]	0.22 (0.824)	0.07 [-0.27, 0.41]	0.41 (0.682)
Time	***0.13*** ***[0.05, 0.21]***	***3.34 (0.001)***	***0.12*** ***[0.06, 0.18]***	***4.11 (<.001)***	***0.15*** ***[0.06, 0.24]***	***3.16 (0.002)***	***0.15*** ***[0.06, 0.24]***	***3.18 (0.002)***
Female sex assigned at birth	0.93 [-1.54, 3.40]	0.74 (0.463)						
Female sex assigned at birth *Time	0.03 [-0.08, 0.14]	0.48 (0.631)						
Gender diversity status			2.75 [-0.86, 6.36]	1.49 (0.137)				
Gender diversity status*Time			0.15 [-0.00, 0.31]	1.95 (0.053)				
Depression History					***7.54*** ***[5.08, 10.00]***	***6.01(<.001)***		
Depression History*Time					7e^-4^ [-0.11, 0.11]	0.01 (0.990)		
Income Level (in $1,000)							***-0.05*** ***[-0.09, -0.00]***	***-2.00 (0.046)***
Income Level*Time							-1.26e^-5^ [-0.001, 0.001]	-0.02 (0.988)
Random Effects	Standard Deviation		Standard Deviation		Standard Deviation		Standard Deviation	
Intercept	10.35		10.33		9.72		10.19	
Time	0.28		0.27		0.27		0.27	
Residual	5.53		5.53		5.53		5.23	

Bold and italicize values are statistically significant: p value <.05.

### Time-varying life circumstance variables

Autistic adults reported lower depressive symptoms when they were engaging in school/work activities. The model-predicted average depressive symptom scores on the DASS-21 were 12.30 (95% CI: 5.35-19.26) when autistic adults were not working or in school, and 1.72 (95% CI: 0.12-3.32) points lower when they were engaged in work or school. There was no interaction between work/school activity and time, indicating that the impact of work/school activity on depressive symptoms did not differ over the course of the study. The random effects in the model revealed between-individual variability in baseline levels of depressive symptoms, changes over time, and the impact of work/school activity on depressive symptoms. The random intercept and random slope of work/school activity were negatively correlated (-0.35), meaning that the effect of work/school activity was stronger among those who started out with higher baseline levels of depressive symptoms. Neither relationship status nor its interaction with time had a significant impact on self-reported depressive symptoms. **Table 4** shows model estimates with the two time-varying variables and their interaction with time.

**Table 4:** Multilevel model results with time-varying variables.

	Work/School Activity		Relationship Status	
Fixed Effects	Estimate (95% CI)	*t value (p)*	Estimate (95% CI)	*t value (p)*
Intercept	12.31 [5.33, 19.29]	3.47 (<.001)	10.49 [3.55, 17.44]	2.97 (0.003)
Age at baseline	0.05 [-0.21, 0.31]	0.40 (0.692)	0.08 [-0.18, 0.34]	0.60 (0.549)
Time	***0.14 [0.05, 0.23]***	***5.16 (<.001)***	***0.16 [0.10, 0.23]***	***4.89 (<.001)***
Work/School Activity	***-1.73 [-3.34, -0.12]***	***-2.47 (0.014)***		
Work/School Activity*Time	0.002 [-0.10, 0.11]			
Relationship status			-0.03 [-1.84, 1.79]	-0.03 (0.977)
Relationship status*Time			-0.06 [-0.17, 0.05]	-1.07 (0.287)
Random Effects	Standard Deviation		Standard Deviation	
Intercept	10.75		10.16	
Time	***0.28***		***0.27***	
Work/School Activity	***3.36***			
Relationship status			2.85	
Residual	5.44		5.50	

Bold and italicize values are statistically significant: p value <.05.

## Discussion

This two-year longitudinal study offers new information to advance our understanding of factors associated with both between-individual differences and within-individual changes in depressive symptoms among autistic adults over time. Autistic adults started the study with relatively high levels of depressive symptoms on average, and symptoms continued to worsen over the course of the study period from March 2020 to March 2022. While these results are consistent with a growing body of literature pointing to depression in autism as a major public health problem, the specific finding of overall worsening depression symptoms must be considered in the specific context of the COVID-19 pandemic. As the pandemic continued to cause prolonged social isolation and life disruption, loss of formal supports or services, and economic uncertainty, autistic adults were likely to experience increased psychological distress (32, 33). Moreover, as the rest of society returned to work, autistic adults possibly faced disparity in (re)gaining employment, though the overall rate of paid employment in this sample remained unchanged (and fairly low: 49%-52% compared to the general population) across all timepoints. Nevertheless, while it is hard to tease apart specific pandemic-related stressors that may have exacerbated depressive symptoms for some, the persistent worsening trend across the two years further underscores the urgent need to understand and treat depression in this population.

Autistic adults reported higher levels of depressive symptoms when they were not engaged in work/school activity, and this was especially pronounced for those with higher depressive symptoms at baseline. Work or school attendance can provide daily structure for autistic adults, with opportunities to participate in goal-directed activities and interact with others in structured settings. These findings highlight the potential utility of treatment targeting life experiences, such as the behavioral activation therapy (34). For example, a cross-sectional study found that autistic adults who are actively engaged behaviorally and socially on a regular basis are more likely to report lower levels of depressive symptoms (5). Additionally, employment may bring more financial security and a sense of productivity and achievement, which are essential to psychological well-being. While the current study only considered paid employment and did not include unpaid work/volunteering (primarily due to extreme variability in volunteer experiences), future studies could investigate whether and how unpaid daytime activities could be leveraged to help meet the social needs of some autistic adults.

Importantly, our findings suggest that the effect of work/school participation varies across individuals, with those with higher baseline depression symptoms experiencing a stronger impact of work/school activities on their depressive symptoms. This finding is in line with previous research showing that individuals with previous psychiatric illnesses are more reactive to distress, especially during a disaster (35). Thus, we need to carefully consider the possibility that individuals with a history of mental health challenges may be even more susceptible to distressing life circumstances (36). In fact, we found that autistic adults with a depression history started with a higher level of depressive symptoms and remained persistently at elevated levels over the two-year study period. It is possible that longer-term follow-up or symptom monitoring starting an earlier age might capture more depression symptom fluctuations, especially in the context of treatment. In addition, future research should focus specifically on understanding the initial onset of depression in autism in order to modify the trajectory of mental health challenges from early on.

The effect of time-varying relationship status was not meaningfully associated with depressive symptoms in the current study. It is important to note that very few individuals reported changes in romantic relationship status (i.e., becoming single or getting into a relationship) across timepoints (less than 10%), limiting our power to detect a possible effect. Changes in romantic partners or the nature of the relationship (e.g., engaged or married) were also not captured in our data. Romantic relationships could have positive (e.g., social connection and support) and negative (e.g., relationship conflicts) effects. Therefore, just being in a relationship might not be as directly relevant to mental health as one’s experience in the relationship. More nuanced indicators of relationship status, including quality of the relationship, are necessary for understanding its impact on mental health.

The current study also examined the effects of time invariant sociodemographic variables that have been shown to influence depression in general population. For instance, to our knowledge, the current study is the first to show that autistic adults with lower income report more depressive symptoms. Given that our study was conducted from 2020-2022, it is also possible that participants with higher income benefitted even more from financial resilience in the face of economic turmoil related to the pandemic, including more access to resources and support to protect them from mental health problems. Further, many autistic adults experience financial challenges due to unemployment and underemployment (37, 38), and they may benefit from income support. Therefore, besides supporting autistic adults through financial hardships, policies promoting employment and living wage might have the added benefit of improving mental health. Notably, the current study did not find a significant effect of sex assigned at birth or diverse gender identity on depressive symptoms. Previous studies have found that autistic women are more likely to be diagnosed with depression (11, 39, 40) and that mental health problems are particularly common in late-diagnosed autistic women (29, 41, 42). It is possible that the finding of no sex differences in our sample is explained by the fact that we only included young adults who had been diagnosed with ASD during childhood.

### Limitations

The current study is among the first to examine longitudinal associations between life circumstances and depressive symptoms in autistic adults. There are several measurement-related limited due the nature of an online survey study. First, we only collected to broad-domain indicators of time-varying factors of work/school participation and romantic relationship status. Specific characteristics of employment, education, and relationship experiences may be more closely associated with psychological well-being. Future research will benefit from incorporating indicators of the quality of employment and school participation and relationships, and autistic adults’ perceptions of and satisfaction with their experiences. Due to limited resources, we could only collect data sporadically to gather snapshots of life experiences, missing possible meaningful changes in between the timepoints. It is imperative to collect more dense longitudinal data to capture life changes as they are happening.

Second, income information was only collected at the last time point, without detailed information on the sources of reported income (e.g., income from work, financial benefits, financial support from families), or whether the income was sufficient to make ends meet. Future studies should collect more comprehensive information about finances in order to better understand associations between financial well-being and mental health in autistic adults. Besides, employment status and income levels are highly confounded. The lack of detailed profiling of the two variables limits our ability to tease apart the relative and interaction effect of income and employment on mental health over time. Importantly, the current investigation of social determinants of mental health is limited due to the lack of sample diversity (50% with some college or above educational level, with one-third still in school, and 80% non-Hispanic White) and likely underestimate the negative impact of mental health disparities that would be observed if the sample was more representative. Future research should consider a more comprehensive characterization of SES and study the intersection of multiple social determinants of mental health in autistic adults. Due to limited sample sizes and the focus on the effect of individual factors, we did not examine the relative effects of different variables nor their interactions. Sociodemographic disparities are observed in vocational, social, recreational, and community participation, such that autistic individuals from more disadvantaged family backgrounds (e.g., lower household income, lower education levels) are less likely to benefit from employment or post-secondary education, participate in social activities, or access needed treatment (8, 43, 44). We believe the current study provides a strong rationale for future studies to investigate the differential impact of influential factors on mental health in autistic adults with diverse sociodemographic backgrounds.

Another limitation is that we did not collect sufficient information on depression treatment to allow examination of the impact of treatment on the trajectory of symptom changes. As many autistic adults desire and value mental health treatment but face service access barriers (45), future research should examine the effect of treatment access and quality on depression in this population. Lastly, while we intentionally only included legally independent adults with a childhood diagnosis of autism, these findings may not generalize to adults with higher support needs who are not capable of consenting or self-reporting independently, nor to adults diagnosed with ASD for the first time in adulthood.

Despite the noted limitations, the current study provides valuable longitudinal data about between-individual differences and within-individual changes in depressive symptoms among autistic young adults over the course of two years. Our findings highlight the need to provide ongoing support for those who experienced depression previously and to carefully attend to the mental health needs of financially disadvantaged individuals. They also point to potential alternative intervention targets, including vocational and educational experiences, to improve mental health.

## Data Availability

The raw data supporting the conclusions of this article will be made available by the authors, without undue reservation.
