# Supplementary material for: Patterns and correlates of two-year changes in depressive symptoms for autistic adults
Source: Front Psychiatry. 2024 Dec 3;15:1461704. doi: 10.3389/fpsyt.2024.1461704 (PMC11650709; doi:10.3389/fpsyt.2024.1461704)
Supplement: Supplementary file 1 [file DataSheet1.docx]

**Supplementary Materials**

**Figure S1 Normality Check for Level 1 Residuals of Depression History and Income Models**


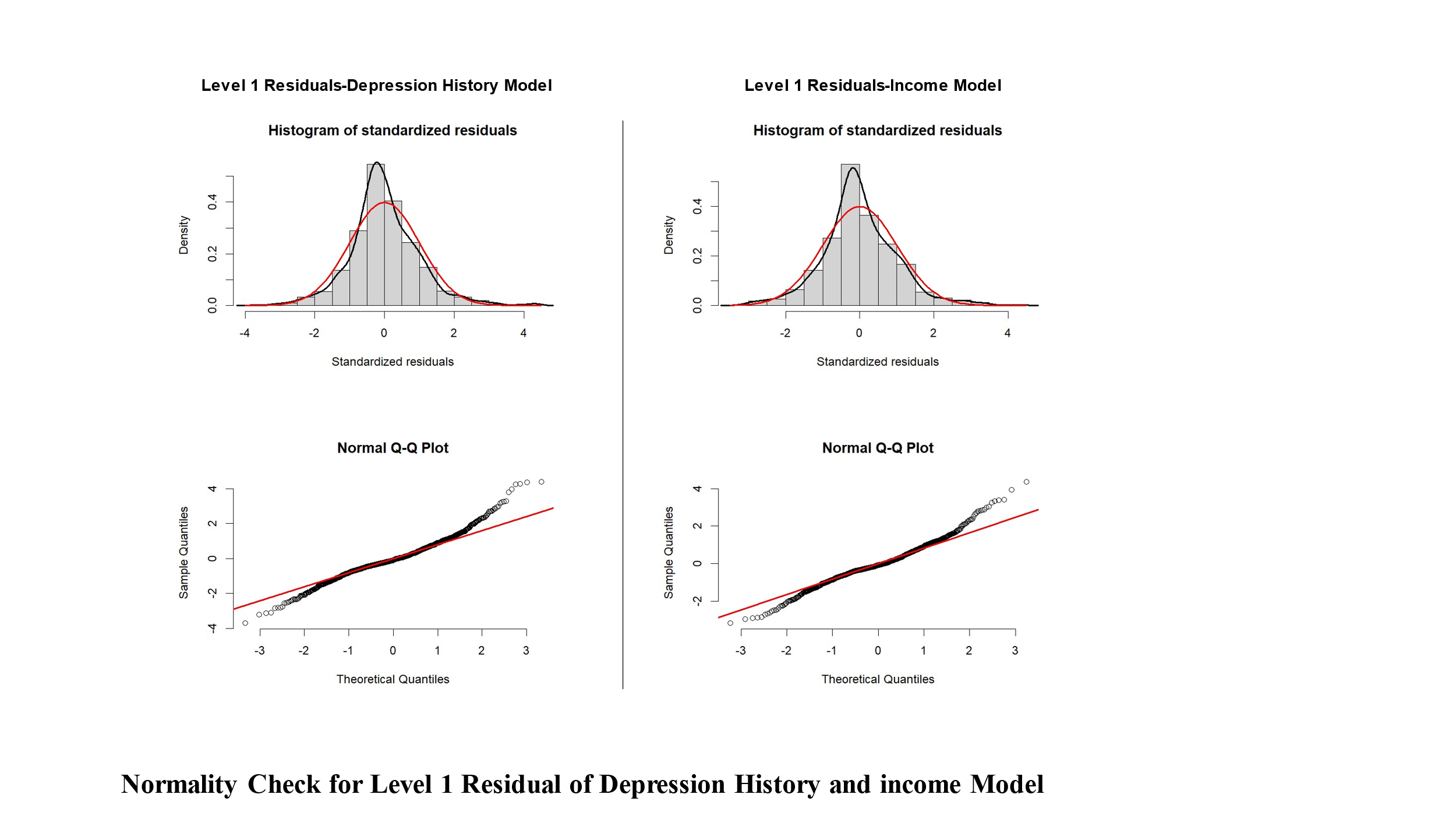


**Figure S2 Normality Check for Work/School Activity Model**


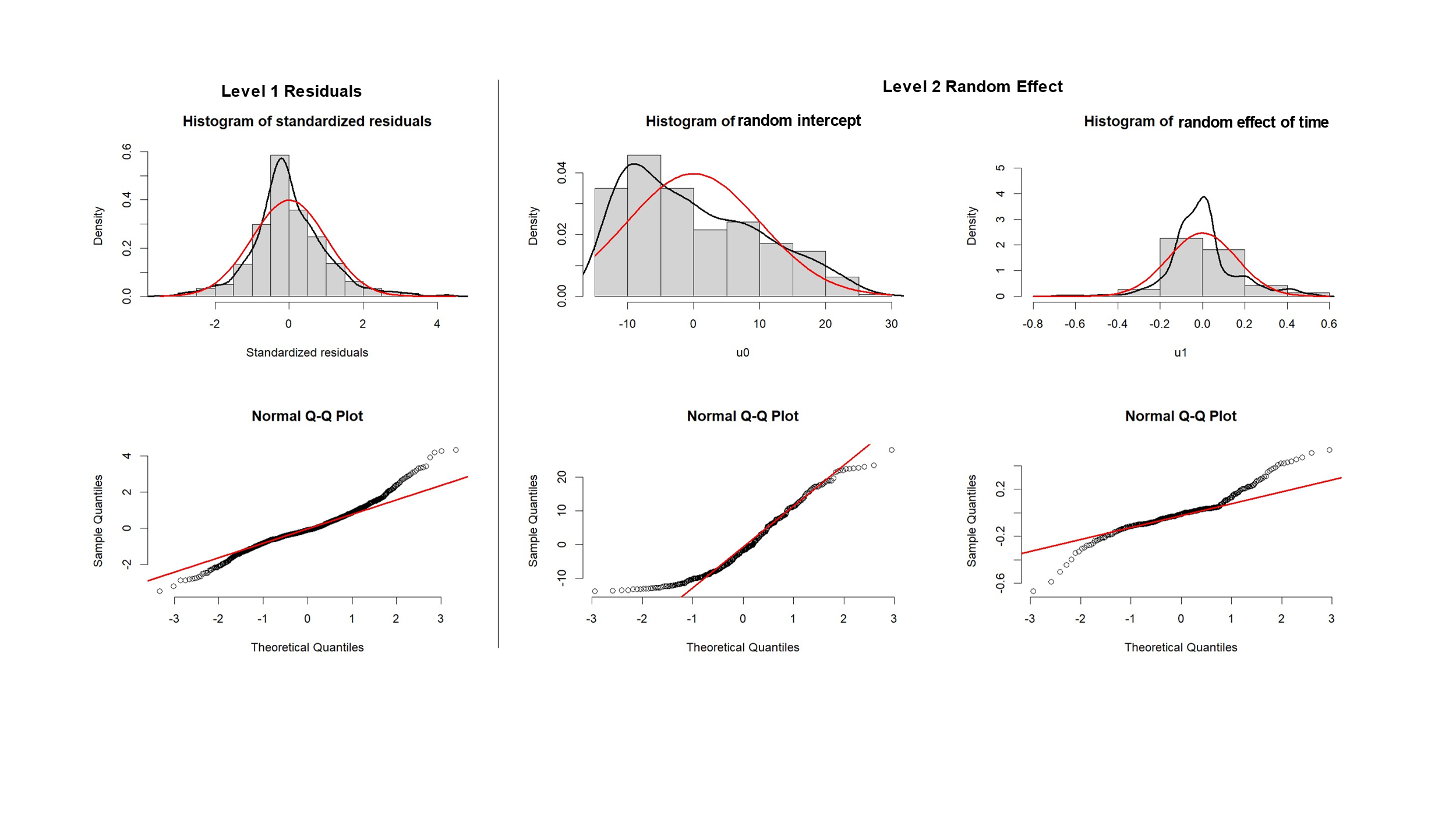


**Table S1 Changes in Work/School Activities and Romantic Relationship Status between Timepoints.**

| **Timepoint** | **Work/School Activities** | | **Romantic Relationship** | |
| --- | --- | --- | --- | --- |
|  | **Gained** | **Lost** | **Gained** | **Lost** |
| Baseline to 05/2020 | 17 (6%) | 27 (10%) | 11 (4%) | 9 (3%) |
| 05/2020 to 03/2021 | 20 (10%) | 11 (5%) | 10 (5%) | 12 (6%) |
| 03/2021 to 07/2021 | 11 (6%) | 14 (7%) | 8 (4%) | 2 (1%) |
| 07/2021 to 03/2022 | 10 (6%) | 18 (11%) | 5 (3%) | 12 (7%) |

**Figure S3 Sample Means Across Timepoint with a Linear Trend Line**


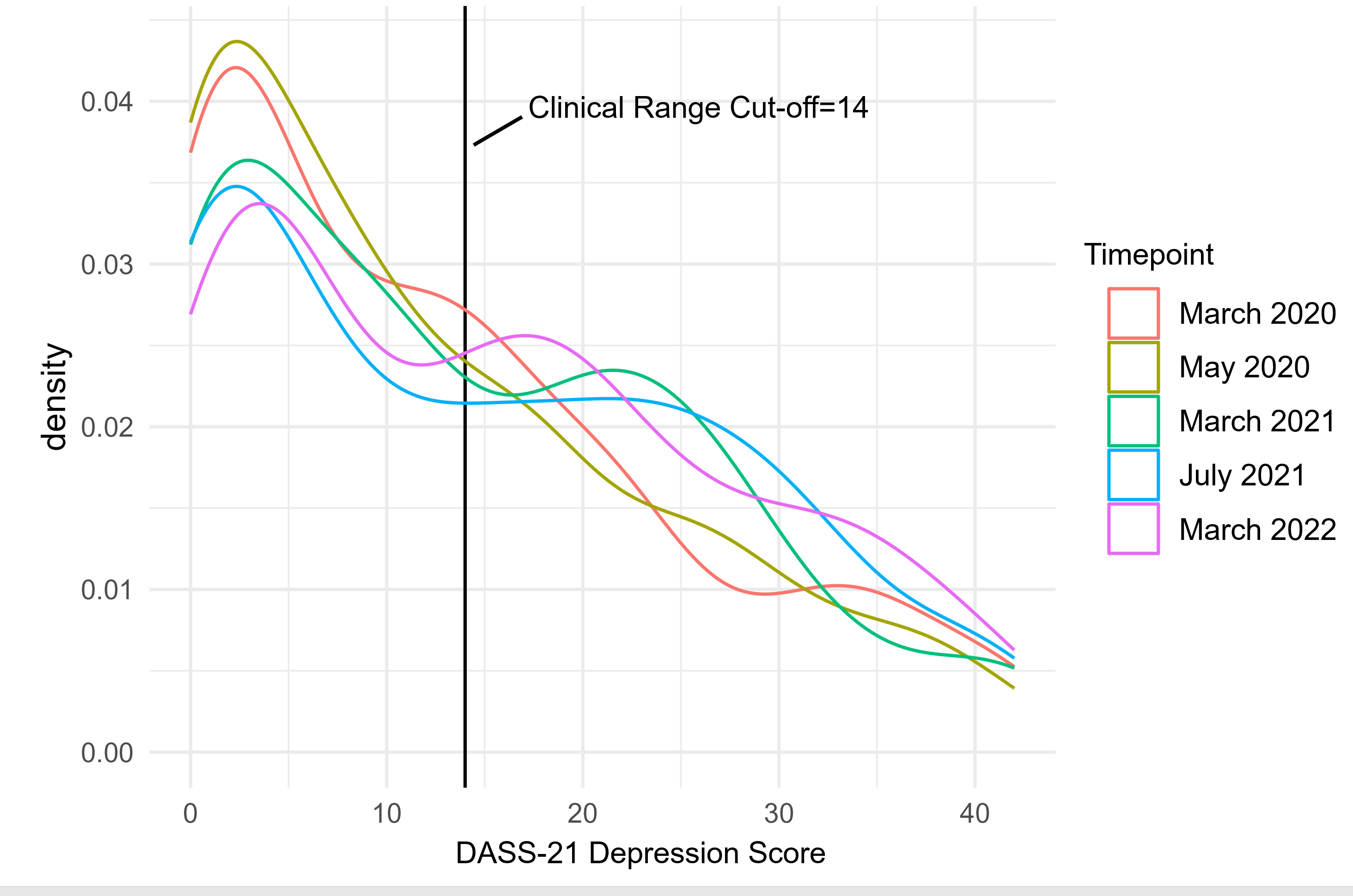


**Figure S4 Proportion of DASS-21 Depression Categories Across Time**
